# The role of transportation in the spread of *Brachyspira hyodysenteriae* in fattening farms

**DOI:** 10.1186/s12917-017-1328-5

**Published:** 2018-01-10

**Authors:** Enrico Giacomini, Sara Gasparrini, Massimiliano Lazzaro, Federico Scali, Maria Beatrice Boniotti, Attilio Corradi, Paolo Pasquali, Giovanni Loris Alborali

**Affiliations:** 1 Istituto Zooprofilattico Sperimentale della Lombardia e dell’Emilia Romagna [Experimental Zooprophylactic Institute of Lombardy and Emilia Romagna] “Bruno Ubertini”, Via Bianchi 7/9, 25124 Brescia, Italy; 2 Department of Veterinary Sciences, University of Parma, Parma, Italy; 3 Istituto Superiore di Sanità, Rome, Italy; 4 FAO Reference Center for Veterinary Public Health, Rome, Italy

**Keywords:** Swine dysentery, *Brachyspira hyodysenteriae*, MLST, MLVA, Transportation, Biosecurity

## Abstract

**Background:**

Direct and indirect contact among animals and holdings are important in the spread of *Brachyspira hyodysenteriae.* The objective of this study was to investigate the role of slaughterhouse vehicles in spreading *B. hyodysenteriae* between unconnected farms.

**Results:**

Multilocus sequence typing (MLST) and Multiple Locus Variable number tandem repeat Analysis (MLVA) were used to characterize *B. hyodysenteriae* strains isolated from trucks. Before cleaning, 976 batches of finishing pigs transported by 174 trucks from 540 herds were sampled. After cleaning, 763 of the 976 batches were also sampled. Sixty-one of 976 and 4 of 763 environmental swabs collected from trucks before and after cleaning and disinfection operations, respectively, were positive for *B. hyodysenteriae*. The 65 isolates in this study originated from 48 farms. Trucks were classified into five categories based on the number of visited farms as follows: category 1: 1–5 farms, category 2: 6–10 farms, category 3: 11–15 farms, category 4: 16–20 farms, category 5: >21 farms. Although the largest number of vehicles examined belonged to category 1, the highest percentage of vehicles positive for *B. hyodysenteriae* was observed in categories 3, 4 and 5. Specifically, 90.9% of trucks belonging to category 5 were positive for *B. hyodysenteriae*, followed by categories 4 and 3 with 85.7% and 83.3%, respectively. The results of MLST and MLVA suggest that trucks transporting pigs from a high number of farms also play a critical role in spreading different *B. hyodysenteriae* genetic profiles. STVT 83–3, which seems to be the current dominant type in Italy, was identified in 56.25% of genotyped isolates. The genetic diversity of isolated strains from trucks was high, particularly, in truck categories 3, 4 and 5. This result confirmed that MLST and MLVA can support the study of epidemiological links between different *B. hyodysenteriae* farm strains.

**Conclusions:**

This study highlights the potential role of shipments in *B. hyodysenteriae* spread. Moreover, it emphasizes the importance of strict vehicle hygiene practices for biosecurity programmes.

## Background

Swine dysentery (SD) is a widely distributed disease that affects pigs in the growing and finishing periods. It can induce high levels of mortality and reduce feed conversion, substantially affecting pig production. The disease’s aetiological agent is *Brachyspira hyodysenteriae*, a gram-negative anaerobic bacterium capable of damaging the enterocytes of the large intestine and causing severe mucohaemorragic enteric disease. Furthermore, *Brachyspira hampsonii*, a strongly hemolytic *Brachyspira* species, was also identified as agent of SD [1, 2]. Nevertheless, *B. hampsonii* was never detected in Italy. The transmission of microorganisms occurs mainly by direct contact with infected pigs and the ingestion of contaminated faeces [1]. Moreover, transmission can follow the introduction of contaminated clothing and footwear of animal caretakers or visitors who have had contact with infected pigs, or it can be conveyed by feral and other animals [1]. Furthermore, motor vehicles used for animal transport seem to be an important potential risk for disease propagation among unconnected farms and slaughterhouses [3]. In fact, vehicles used in animal transportation can be involved in the indirect spread of infectious agents that may cause disease, such as foot and mouth disease (FMD) [4], porcine reproductive respiratory syndrome (PRRS) [5, 6] and porcine epidemic diarrhoea (PED) [7]. It was observed that when animals from different farms are transported separately but in successive shipments, the risk of transmission of infectious agents potentially increases [8, 9]. Therefore, appropriate cleaning and the application of a disinfection protocol for shipment vehicles, as well as biosecurity measures at the farm gate, may be crucial for limiting the spread of disease via shared trucks [10].

In this study, we aimed to investigate the role of trucks in spreading *B. hyodysenteriae* between unconnected farms. To better understand the route of transmission and the source of SD outbreaks, we sampled slaughterhouse vehicle protocols and characterized *B. hyodysenteriae* strains isolated from trucks by combining multilocus sequence typing (MLST) and multiple locus variable number tandem repeat analysis (MLVA).

## Methods

### Sample collection

A batch of finishing pigs, sent to slaughter by truck, was considered a single epidemiological unit. Between 2012 and 2015, environmental swabs were sampled from trucks for 976 selected epidemiological units. A total of 174 trucks were used to transport those units, which were from 540 different herds. Environmental swabs were collected on trucks at the staging place of the slaughterhouse. Each sample was collected by swabbing the four corners and the perimeter of the low floor of the trucks. One swab per truck was collected before sanitizing procedures and, when possible, one swab was taken after such procedures. Each sample was placed in a single sterile bag, stored in a refrigerated container, and processed within 5 h. Before the cleaning and disinfection operations, 976 samples from 174 trucks were collected. After routine cleaning and disinfection, 763 environmental swabs were taken from 147 of the 174 trucks examined. The procedures applied at the slaughterhouses required that after unloading the animals, the trucks were washed with high-pressure washing systems and disinfected with products based on glutaraldehyde, quaternary ammonium salts, chloramine T and Virkon®.

### *B. hyodysenteriae* isolates

Swab samples kept in sterile bags were washed with 50 ml of saline solution. The liquid was then transferred to a sterile tube and clarified by centrifugation for 5 min at 1500×g. DNA extraction was then performed with 1 ml of the supernatant of each environmental sample using a DNeasy Blood & Tissue Kit (Qiagen GmbH, Germany). The bacterial DNA was eluted into 100 μl of elution buffer. A species-specific real-time PCR assay was performed to detect the presence of *B. hyodysenteriae, B. pilosicoli* and *Lawsonia intracellularis* DNA [11].

Using a pad saturated with washing liquid, each PCR-positive sample was seeded on blood agar culture plates supplemented with rifampicin, vancomycin HCl and colistin and incubated at 37 °C in an anaerobic atmosphere for 5 days. The evaluation of their haemolytic characteristics (strong beta haemolysis) allowed for the identification of samples positive for *B. hyodysenteriae.* Bacterial cells with strong haemolysis were then harvested and resuspended in TE buffer (300 μl). DNA was obtained by boiling the samples at 98 °C for 10 min, and the DNA was stored at 4 °C. A species-specific PCR assay was performed to confirm the classification of *B. hyodysenteriae* or *B. pilosicoli* [11].

### Genotyping

The obtained *B. hyodysenteriae* pure isolates were then characterized using MLST and MLVA. MLST was performed on seven loci coding for housekeeping genes [12, 13]. We used the same PCR conditions and purification and PCR sequencing methods as previously described [14]. The sequences obtained were then analysed and codified to obtain a sequence type (ST) [13]. MLVA was conducted by analysing seven multiple variable number tandem repeats (VNTR) loci within the genome using the primers described by Hidalgo and colleagues [15] and the PCR conditions described by Gasparrini and colleagues [14]. The amplicons were analysed on a 2% agarose gel to estimate the locus size and calculate the number of repeats. Hence, the MLVA profiles (VT) were codified [15].

To better characterize the isolates collected, MLST and MLVA profiles were combined (STVT profiles) as previously illustrated [14].

### Truck categorisation

The trucks transporting the finishing pig batches were identified and associated with the farms from which the animals originated. The trucks were classified into different categories based on the number of farms visited as follows: category 1: 1–5 farms visited, category 2: 6–10 farms visited, category 3: 11–15 farms visited, category 4: 16–20 farms visited, category 5: >21 farms visited. The percentage of vehicles positive for *B. hyodysenteriae* and the MLST and MLVA profiles of *B. hyodysenteriae* isolates were evaluated according to truck categories.

### Statistical analysis

The statistical analysis was conducted by considering two groups of trucks: vehicles transporting pigs from up to five different farms and trucks visiting more than five farms. Differences in the prevalence of positive samples between the two groups were estimated with a chi-square test and odds ratio (OR) with a 95% confidence interval (CI). All statistical computations were performed with GraphPad Prism 6 (GraphPad Software, San Diego, CA, USA). The significance level was set at *P* < 0.05.

## Results

### Samples

Before cleaning and disinfection operations, 976 environmental samples, one for each batch of pigs, were collected. After the sanitisation routine, 763 of the 976 sampled batches (78.18%) were also sampled.

The real-time PCR analysis performed on the environmental samples collected from trucks before the cleaning and disinfection operations showed that 61 of 976 environmental swabs were positive for *B. hyodysenteriae* (6.25%, Table 1). Four of the 763 environmental swabs collected after routine cleaning and disinfection operations were positive for *B. hyodysenteriae* (0.52%). All four positive samples were collected from trucks where swabs were also positive before cleaning. Two trucks each from categories 4 and 5 had positive samples. Moreover, two of the four positive samples were collected the same day at the same slaughterhouse. Overall, the 65 *B. hyodysenteriae* isolates detected originated from 48 (8.89%) of the 540 tested farms (Table 1).

**Table 1:** Yearly distribution of total and real-time PCR-positive environmental swabs collected from trucks before and after cleaning and disinfection operations

Year	Before cleaning			After cleaning			Farms^a^		
	Total	Positive	%	Total	Positive	%	Total	Positive	%
2012	247	6	2.43%	174	0	0.00%	134	6	4.48%
2013	110	1	0.91%	86	0	0.00%	72	1	1.39%
2014	89	4	4.49%	76	2	2.63%	70	4	5.71%
2015	530	50	9.43%	427	2	0.47%	264	37	14.02%
TOT	976	61	6.25%	763	4	0.52%	540	48	8.89%

^a^Number of farms from which the pigs transported by trucks originated

### Truck categories

The finishing pig batches were transported using 174 trucks classified into 5 categories based on the number of farms visited, as described in the Methods section.

**Fig. 1:**
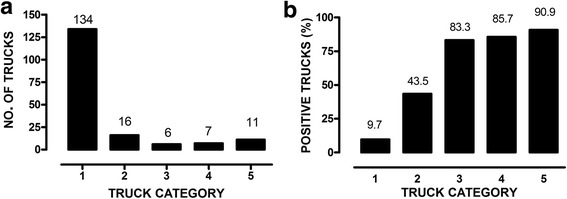
Distribution of vehicles transporting pigs (**a**) and *B. hyodysenteriae* positive vehicles (**b**) separated by truck categories. Most of the vehicles belonged to category 1, but most of the positive samples were detected in categories 3–5

In particular, the largest number of vehicles examined belonged to category 1 (*n* = 134). Sixteen trucks were in category 2, 6 trucks were in category 3, 7 trucks were in category 4, and 11 trucks were in category 5 (Fig. 1a). Overall, 22.99% of the trucks were positive for *B. hyodysenteriae* with at least 1 swab.

As shown in Fig. 1b, 90.9% of trucks belonging to category 5 were positive for *B. hyodysenteriae*, followed by truck categories 4 and 3 with 85.7% and 83.3%, respectively. *B. hyodysenteriae* was detected in 7 of the 16 trucks (43.5%) belonging to category 2, and only 13 trucks belonging to category 1 were positive (9.7%).

### Genotyping

Sixteen pure isolates of *B. hyodysenteriae* were obtained from the 65 *B. hyodysenteriae* PCR-positive samples collected from different trucks. It was not possible to isolate any strains from 33 *B. hyodysenteriae* PCR-positive swabs, and 16 PCR-positive samples presented a *Brachyspira* spp. mixed culture. The 16 pure isolates, which originated from swab collected before sanitation procedures, were analysed using MLST and MLVA, and the genetic profiles obtained were combined. Eight MLST-MLVA profiles were identified. Table 2 shows the MLST and MLVA profiles of *B. hyodysenteriae* strains isolated from vehicles at slaughterhouses in relation to the 5 truck categories. Thirteen of the 16 isolates were detected in truck categories 3–5, with a high number of farms involved for each vehicle. The most frequent profile was 83–3, found in trucks belonging to all the categories, while several other profiles were isolated in only a single sample (Table 2).

**Table 2:** MLST and MLVA profiles of the 16 isolates from vehicle swabs in relation to truck category. All 16 isolates were obtained from swabs collected before cleaning and disinfection operations. The second row displays the sequence type (ST) of each isolate, and the third row displays the variable number tandem repeats type (VT)

Isolate number	1	2	3	4	5	6	7	8	9	10	11	12	13	14	15	16
ST	75	83	83	206	83	83	83	83	76	209	205	83	83	74	83	79
VT	11	3	3	18	3	3	3	3	6	22	2	3	3	2	3	9
Truck category	4	5	4	5	1	3	1	1	3	3	3	4	2	5	5	5

**Fig. 2:**
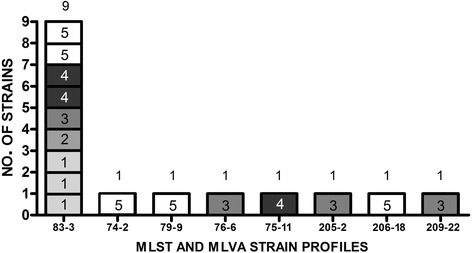
Number of MLST and MLVA strain profiles isolated from different truck categories. Nine of 16 isolates belonged to the STVT 83–3 profile, while the remaining isolates had different profiles. Each box represents one isolate, and the number within a box represents the category of the source truck

MLST and MLVA strain profiles isolated from trucks are reported in Fig. 2.

## Discussion

The results of the investigation of the environmental samples collected from trucks before cleaning and disinfection operations showed that 6.25% of environmental swabs and 8.89% of farms were *B. hyodysenteriae* positive. These data suggest that transportation can play a potential important role in *B. hyodysenteriae* spreading between otherwise unconnected farms and that it can be a critical point for SD control.

Very limited information is available on the role of trucks in spreading SD. It is known that new outbreaks of SD usually occur following introduction of asymptomatic carrier pigs into uninfected herds, by contaminated feed or animal trucks, or by rodents or visitors who have come into contact with infected pigs [4, 16]. The role of the transportation equipment used to move pigs is well known to spread other diseases, such as porcine epidemic diarrhoea virus (PEDV), transmissible gastroenteritis virus (TGEV) [7] and porcine reproductive respiratory syndrome virus (PRRSV) [6].

The results of our investigations suggest that trucks are involved in *B. hyodysenteriae* spread and that trucks that visit a higher number of farms will have a greater potential of testing positive for *B. hyodysenteriae*. Overall, the percentage of positive samples was significantly higher (*P* < 0.0001) in trucks that visited more than five farms than in trucks that visited fewer than 5 farms (OR = 21.72, 95% CI 8.95–52.67).

The sharing of trucks between farms for the shipment of pigs may increase the risk of disease propagation [8] due to poor cleaning and disinfection of these trucks [17, 18].

The low number of *B. hyodysenteriae*-positive samples (0.52%) after cleaning and disinfecting procedures should not be neglected. The frequency of positive samples after cleaning and disinfecting procedures may be underestimated. Indeed, because of truckers’ tight schedules, it was not possible to sample 213 of the 976 shipments (21.82%) after the cleaning routine. It is possible that a transporter who is in a hurry has a higher risk of overlooking some parts of the cleaning and disinfecting routines. All positive samples came from trucks in category 4 or 5, which visited a high number of farms (*n* = 47). Furthermore, two of the four positive samples were collected the same day at the same slaughterhouse, suggesting at least a partial failure of the sanitisation process. *B. hyodysenteriae* is relatively resistant in moist faeces, and it survived for 48 days at 0–10 °C, 7 days at 25 °C, and less than 24 h at 37 °C [19]. It survived at 10 °C for 78 days in soil with 10% pig faeces and for 112 days in pure pig faeces [20]. Moreover, Lambert and colleagues reported that less than one-third of the trucks used for the shipment of pigs in Canada are cleaned and disinfected between successive shipments [18]. Percentage of trucks with inadequate sanitation may differ in the working conditions in which the current study was performed. However, considering that 22.9% of trucks sampled in this study transported at least one *B. hyodysenteriae-*positive batch, shipments may still play an important role in spreading the pathogen among unconnected herds, particularly when farms do not follow strict external biosecurity measures. On the other hand, 33 out of 65 PCR-positive samples were impossible to isolate suggesting that either a low bacterial load was collected or bacteria viability was already reduced before swabbing. Particularly, in the latter case, viability of bacteria to infect animals could be reduced or absent.

MLST and MLVA profile results suggest that trucks transporting pigs from a high number of farms may also play a potential role in spreading different *B. hyodysenteriae* genetic profiles. In general, the same strains isolated in trucks were found in high production areas, as previously reported by Gasparrini and colleagues [14]. Nevertheless, the role of these trucks in spreading different *B. hyodysenteriae* strains should be further investigated because one of the limitations of this study is that only one isolate per batch was identified.

The STVT 83–3 profile, present in 9 of 16 samples, should be the current dominant type in Italy. In two recent studies, STVT 83–3 represented more than 45% of the *B. hyodysenteriae* isolates from 2012 to 2015 [14, 21]. Notwithstanding the low number of isolated strains from trucks, the strain genetic diversity is high. In fact, 8 genetic profiles in 16 isolates were found in the trucks (ratio = 0.5), while 33 of 152 MLST and MLVA profiles were found in the farms (ratio = 0.21) [14], highlighting the role of transportation in collecting and spreading many different *B. hyodysenteriae* strains. The genetic profiles isolated from trucks, but not from outbreaks, belonged to truck categories 3, 4 and 5. This result confirmed that MLST and MLVA can support the study of epidemiological links between different *B. hyodysenteriae* farm strains [22].

## Conclusions

Despite its limitations, this study suggests that *B. hyodysenteriae* control measures should include a more stringent application of standardized procedures for hygiene practices to improve the sanitation of vehicles at slaughterhouses. These control measures may be implemented at collection points and slaughterhouses to prevent contaminated vehicles from returning to pig farms and most likely play a role in spreading *B. hyodysenteriae* across vast geographic areas. However, other measures at the farm level, such as loading pigs outside the premises and limiting contact between farmers and trucks during the unloading process, should have a positive effect in reducing the transmission of *B. hyodysenteriae*. Biosecurity measures, at truck and farm levels, are pivotal and require a coordinated effort between producers, transporters and slaughter facility owners to achieve effective implementation. This study on *B. hyodysenteriae* transmission should provide more information regarding the role of shipments, and it highlights the risks that may occur if coordinated biosecurity programmes, which involve finisher transportation, are not properly implemented.

## Abbreviations

CI: Confidence interval
FMD: Foot and mouth disease
MLST: Multilocus sequence typing
MLVA: Multilocus variable-number tandem-repeat analysis
OR: Odds ratio
PCR: Polymerase chain reaction
PED: Porcine epidemic diarrhoea
PEDV: Porcine epidemic diarrhoea virus
PRRSV: Porcine reproductive and respiratory syndrome
PRRSV: Porcine reproductive and respiratory syndrome virus
SD: Swine dysentery
ST: Sequence type
STVT: Sequence type and variable number tandem repeats type
TGEV: Transmissible gastroenteritis virus
VNTR: Variable number tandem repeats
VT: Multiple variable number tandem repeats type
